# The burden of co-existing dermatological disorders and their tendency of being overlooked among patients admitted to muhimbili national hospital in Dar es Salaam, Tanzania

**DOI:** 10.1186/1471-5945-11-8

**Published:** 2011-04-14

**Authors:** Yassin M Mgonda, Pauline NF Chale

**Affiliations:** 1 Department of Internal Medicine, Muhimbili University of Health and Allied Sciences, Dares Salaam, Tanzania

## Abstract

**Background:**

Skin diseases are underestimated and overlooked by most clinicians despite being common in clinical practice. Many patients are hospitalized with co-existing dermatological conditions which may not be detected and managed by the attending physicians. The objective of this study was to determine the burden of co-existing and overlooked dermatological disorders among patients admitted to medical wards of Muhimbili National hospital in Dar es Salaam.

**Study design and settings:**

A hospital-based descriptive cross-sectional study conducted at Muhimbili National hospital in Dar es Salaam, Tanzania.

**Methods:**

Patients were consecutively recruited from the medical wards. Detailed interview to obtain clinico-demographic characteristics was followed by a complete physical examination. Dermatological diagnoses were made mainly clinically. Appropriate confirmatory laboratory investigations were performed where necessary. Data was analyzed using the 'Statistical Package for Social Sciences' (SPSS) program version 10.0. A p-value of < 0.5 was statistically significant.

**Results:**

Three hundred and ninety patients admitted to medical wards were enrolled into the study of whom, 221(56.7%) were females. The mean age was 36.7 ± 17.9 (range 7-84 years). Overall, 232/390 patients (59.5%) had co-existing dermatological disorders with 49% (191/390) having one, 9% (36/390) two and 5 patients (1%) three. A wide range of co-existing skin diseases was encountered, the most diverse being non-infectious conditions which together accounted for 36.4% (142/390) while infectious dermatoses accounted for 31.5% (123/390). The leading infectious skin diseases were superficial fungal infections accounting for 18%. Pruritic papular eruption of HIV/AIDS (PPE) and seborrheic eczema were the most common non-infectious conditions, each accounting for 4.3%. Of the 232/390 patients with dermatological disorders, 191/232 (82.3%) and 154/232 (66.3%) had been overlooked by their referring and admitting doctors respectively.

**Conclusion:**

Dermatological disorders are common among patients admitted to medical wards and many are not detected by their referring or admitting physicians. Basic dermatological education should be emphasized to improve knowledge and awareness among clinicians.

## Background

Skin diseases are diverse in clinical presentation, severity and epidemiology although dermatology may simply be considered by many as an outpatient service with very little attention given to admitted patients. Data from several studies on the overall community-based prevalence of skin diseases in developing countries indicate that they are quite common, ranging from 20% to 80% and most of them result from infections such as superficial mycoses, pyodermas and scabies [1-4]. Dermatological disorders affect all age groups of both sexes, however, children are more vulnerable and have been extensively studied worldwide [1,5-8]. The fact that dermatological disorders are very common in the general population and therefore, expected to be common even among hospitalized patients should not mean that they are of less medical importance than other systemic diseases.

There are, but relatively few studies which have documented on dermatological disorders in hospitalized patients [9-13]. The prevalence of dermatological disorders among admitted patients reported by some of these studies shows an almost similar spectrum, differing only in magnitude between developing and developed countries [14]. Conditions such as Stevens Johnson syndrome (SJS), toxic epidermal necrolysis (TEN), autoimmune blistering diseases, necrotizing fasciitis, leg ulcers, dermatological malignancies and psoriasis have been reported as the common indications for hospital admission in developing as well as the developed countries[14,15].

Reports from other studies rarely mention dermatological disorders as a primary reason for hospital admission. Most skin diseases may be overlooked by health professionals despite being important indicators of some underlying internal diseases. Skin diseases may coincidentally co-exist with other medical illnesses or be specific markers/manifestations of underlying systemic diseases. Some of the systemic diseases that can be suspected through cutaneous manifestations include chronic renal failure, endocrine disorders, lymphomas, nutritional deficiencies and HIV/AIDS. The HIV/AIDS pandemic has increased the number of hospital admissions worldwide, particularly in sub Saharan Africa and has been associated with a wide range of dermatological syndromes [16-18]. Cutaneous malignancies, candidiasis, dermatophytoses, molluscum contagiosum, seborrheic dermatitis and pruritic papular eruption have been frequently reported in individuals with HIV/AIDS, seen as inpatients or outpatients [19]. The objective of this study was therefore to describe the burden of co-existing dermatological disorders among patients admitted to medical wards of Muhimbili National hospital in Dar es Salaam and document the tendency if any, of being overlooked by their attending clinicians.

## Methods

### Study design and setting

This was a hospital-based cross sectional descriptive study conducted in the medical wards of Muhimbili National Hospital in Dar es Salaam, Tanzania. Muhimbili National Hospital is the largest tertiary referral and teaching hospital receiving patients from all regions and Dar es Salaam is the largest city in the country.

### Clinical work up

Patients referred by medical officers (MD) from peripheral hospitals all over the country to Muhimbili national hospital's medical wards, were consecutively recruited into the study. Patient's files were systematically scrutinized to obtain useful demographic and clinical data. The referral dermatological diagnoses were retrieved from the patient's referral medical notes. Complete physical examination was conducted at admission by the admitting medical officer who made the 'dermatological diagnosis at admission'. These diagnoses were finally confirmed by one of the three dermatologists based in the hospital, and where necessary consultative discussions were held to make a common final diagnosis. The majority of the dermatological diagnoses were made clinically and where necessary and feasible, appropriate laboratory investigations were arranged.

### Laboratory procedures

Appropriate specimens were collected and sent to the general pathology laboratory (since there is no specialized dermatology laboratory in the hospital) for bacteriological, mycological, parasitological or histo-pathological studies as appropriate. Specimens from suspected fungal lesions underwent microscopic examination after treatment with 10% potassium hydroxide solution. Skin scrapings from scabies lesions were obtained from burrows and examined for mites under a light microscope with a drop of normal saline. Skin biopsy specimens were placed in bottles containing 10% buffered formalin before being stained with Haematoxylin and Eosin and then mounted on Dextrene Polystyrene Xylene (DPX), followed by microscopic examination.

### Data analysis

Data analysis was done using statistical package for social sciences (SPSS) computer program version 10.0. P-value less than 0.05 was regarded as significant.

### Ethical considerations

Ethical clearance was obtained from the ethical clearance board of the Muhimbili University of Health and Allied Sciences (MUHAS). Permission to conduct the study in the medical wards of Muhimbili National Hospital (MNH) was sought from the hospital authority. Fully informed verbal and written consent was obtained from every patient who participated in the study.

## Results

A total of 390 patients admitted to medical wards were recruited into the study. The demographic characteristics and prevalence of dermatological disorders among these patients has been shown in table 1. The mean age was 36.7 years (SD ± 17.9) with the majority (52.3%) being young adults aged 18 - 44 years and 56.7% (221/390 were females. Overall, 232/390 patients (59.5%) had co-existing dermatological disorders with 49% (191/390) having one, 9% (36/390) two and 5 patients (1%) three. Patients in age groups 18-31 years (70%) and 32-44 years (66%) had the highest prevalence than the other age groups (p = 0.01 and 0.04 respectively).

**Table 1:** The prevalence of dermatological disorders among patients admitted to medical wards distributed by age and sex (n = 390)

Variable	All patients n (%)	Patients with dermatological disorder n(%)	OR (95% CI	P-value
**Sex**				
Female	221 (56.7)	122 (55.2)	1.0	0.05
Male	169 (43.3)	110 (65.1)	1.51 (0.99-2.29)	
				
**Age (years)**				
**7-17**	59 (15.1)	29 (49.2)	1.0	
**18-31**	105 (26.9)	73 (69.5)	2.36 (1.20-4.63)	0.01
**32-44**	99 (25.4)	65 (65.7)	1.98 (1.01-3.86)	0.04
**45-60**	80 (20.5)	41(51.3)	1.09 (0.55-2.14)	0.81
**61-84**	47 (12.1)	24 (51.1)	1.08 (0.49-2.33)	0.85
**Total**	**390 (100)**	**232 (59.5)**	-	-

Table 2 shows the dermatological disorders among patients admitted to medical wards. A wide range of skin diseases was encountered, the most diverse being non-infectious conditions which put together accounted for 36.4% (142/390), while infectious dermatoses accounted for 31.5% (123/390). The common non infectious skin diseases were pruritic papular eruption of HIV/AIDS (PPE) present in 4.3% (17/390), seborrheic dermatitis in 3.8% (15/390), non specific ulcers in 3.8% (15/390) followed by acne vulgaris and keloids, all present in 3%. The common infectious dermatoses were superficial fungal infections at a prevalence of 18% (70/390) with dermatophytoses (tinea) accounting for 10%, pityriasis versicolor 4.6% and oral thrush 3.3%. The commonest dermatophytoses were tinea corporis and tinea unguium both with prevalence of 3.6%. Bacterial skin infections (pyodermas) were seen in 7% (28/390), viral infections in 5% and 4 patients (1%) had scabies. Kaposi's sarcoma was the commonest malignancy observed in 11/390 (2.8%) of the 12 patients with cutaneous neoplasms. One patient had squamous cell carcinoma.

**Table 2:** Dermatological disorders among patients admitted to medical wards (n = 390)

Dermatological disorder	n(%)
**I: Non-infectious dermatological disorder**	**142 (36.4)**
-Pruritic Papular Eruption of HIV (PPE)	17 (4.3)
-Seborrheic dermatitis	15 (3.8)
-Non-specific ulcers	15 (3.8)
-Acne vulgaris	12 (3.0)
-Keloids	12 (3.0)
-Prurigo nodularis chronicus	9 (2.3)
-Keratosis plantaris climactericum	8 (2.0)
-Atopic eczema	6 (1.5)
-Psoriasis	5 (1.2)
-Xerosis	4 (1.0)
-Lymphedema	4 (1.0)
-Lichen planus	3 (0.7)
-Stevens-Johnson syndrome	3 (0.7)
-Vitiligo	3 (0.7)
-Fixed drug eruption	3 (0.7)
-Lichen simplex chronicus	2 (0.5)
-Papular urticaria chronicus	2 (0.5)
-Bullous pemphigoid	1 (0.2)
-Scleroderma	1 (0.2)
-Albinism	1 (0.2)
-Non-specific dermatitis	12 (3.0)
**II: Infectious dermatological disorder**	**123 (31.5)**
**(a) Fungal infections (Dermatophyte + Yeast infections)**	**70 (18)**
• **Dermatophyte infections/tinea**	**39 (10)**
-Tinea corporis	14 (3.6)
-Tinea unguium	14 (3.6)
-Tinea pedis	7 (1.8)
-Tinea cruris	3 (0.8)
-Tinea capitis	2 (0.5)
• **Yeast infections**	**31 (8.2)**
-Pityriasis versicolor	18 (4.6)
-Oral candidiasis	13 (3.3)
**(b) Bacterial infections**	**28 (7.1)**
-Infected ulcers	10 (2.5)
-Erythrasma	8 (2.0)
-Cellulitis	6 (1.5)
-Furuncles/carbuncles	3 (0.8)
-Superficial folliculitis	2 (0.5)
-Impetigo	2 (0.5)
**(c) Viral infections**	**21 (5.3)**
-Common warts	13 (3.3)
-Herpes simplex	5 (1.3)
-Genital warts	2 (0.5)
-Chicken pox	2 (0.5)
-Molluscum contagiosum	1 (0.2)
-Herpes zoster	1 (0.2)
**(d) Parasitic infestations**	**4 (1.0)**
-scabies	4 (1.0)
**III: Neoplatic dermatological disorders**	**12 (3.0)**
-Kaposi's sarcoma	11 (2.8)
-Squamous cell carcinoma	1 (0.2)
**Total**	**232 (59.5)**

Table 3 shows the pattern of dermatological disorders as detected at pre-admission, admission and post-admission periods. Out of 390 admitted patients, 41(10.5%) were reported as having dermatological disorders pre-admission, with Kaposi's sarcoma being over diagnosed in 3 patients giving a false prevalence of 3.6% against the actual prevalence of 2.8%. At admission, 20% were reported as having dermatological disorders, the leading diagnoses being fungal infection in 5.1%. Kaposi's sarcoma was reported in 15 patients (3.8%) being an over diagnosis by 4 patients. In the post-admission period when patients were thoroughly reviewed and dermatological diagnoses established, confirmed or ruled out, a total of 232/390 (59.5%) patients were found to have dermatological disorders. In general, of the 232/390 patients with dermatological disorders, 191/232 (82.3%) and 154/232 (66.3%) had been overlooked by the referring and admitting doctors respectively.

**Table 3:** Dermatological disorders diagnosed at pre admission, admission and post admission periods among patients admitted to medical wards (n = 390).

	Time of diagnosis		
Dermatological disorder	Pre-admission n(%)	Admission n(%)	Post-admission n(%)
**All types**	**41(10.5)**	**78 (20)**	**232 (59.5)**
**I: Non-Infectious disorders**	**36 (9.2)**	**52 (13.3)**	**142 (36.4)**
-Inflammatory dermatoses*	12 (5.6)	23 (3.8)	58 (15)
-Kaposi's sarcoma	14 (3.6)	15 (3.8)	11 (3)
-Squamous cell carcinoma	0	0	1 (0.3)
-Nonspecific lesions	10 (2.6)	14 (3.6)	72 (18.2)
			
**II: Infectious disorders**	**5 (1.3)**	**35 (9)**	**123 (31.5)**
-Fungal infections	2 (0.5)	20 (5.1)	70 (18)
-Bacterial infections	2 (0.5)	8 (2.0)	28 (7.1)
-Viral infections	1 (0.2)	7 (1.8)	21 (5.3)
-Mite Infestation	0	0	4 (1.0)
- (Scabies)			

*includes: eczemas, papulosquamous disorders, primary blistering diseases, ulcers, Keloids and connective tissue/autoimmune diseases

Table 4 shows the pattern of co-existence between dermatological disorders and other medical diseases. Overall, 133/390 (34.1%) patients were admitted due to infectious diseases and of these, 82% (109/133) had co-existing dermatological disorders. The infectious diseases which had co-existing dermatological disorders were: HIV disease (86/109; 79%), tuberculosis (13/109; 12%), malaria (7/109; 6.4%), meningitis, (2/109; 2%) and pneumonia (1/109; 1%). Dermatological disorders which mostly co-existed with HIV disease included Kaposi's sarcoma (11/11; 100%), PPE (15/17; 88.2%), oral candidiasis (11/13; 8.6%), psoriasis (4/5; 80%) common warts (10/13; 77%), and seborrheic dermatitis (10/15; 66.7%).

**Table 4:** The frequency of co-existence of dermatological disorders with systemic diseases among patients admitted to medical wards (n = 390)

Systemic disease	Frequency among Admitted patients n(%)	Frequency of co-existing dermatological disorders n(%)
Infectious diseases	133 (34.1)	109 (82)
Cardiovascular diseases	75 (19.2)	33 (44)
Hematological diseases	55 (14.1)	24 (43.6)
Diabetes mellitus	33 (8.5)	22 (66.7)
Renal diseases	29 (7.4)	19 (55.1)
Hepatic diseases	19 (4.9)	12 (63.1)
Solid malignancies	26 (6.7)	12 (46.1)
Neurological diseases	14 (3.6)	8 (57.1)

Thirty three patients were admitted due diabetes mellitus and 22 of them (66.7%) had co-existing dermatological disorders commonly tinea lesions 45.4% (10/22) and ulcers 18.1% (4/22). Among 75/390 (19.2%) patients with cardiovascular diseases, 33 (44%) had co-existing dermatological disorders. Fifty five patients (14%) were admitted due to hematological diseases and 43.6% (24/55) had co-existing dermatological disorders: leukemia (9/24; 37.5%), sickle cell disease (9/24; 37.5) and anemia (6/24; 25%). Among 29 (7.4%) patients with renal diseases, 16 (55.1%) had co-existing dermatological disorders while of the 19 patients (4.9%) admitted due to liver diseases, 12 (63.1%) had co-existing dermatological disorders, mostly xerosis generalisata and prurigo simplex. Twenty six patients (6.7%) were admitted due to solid malignancies and of these, 12 (46.1%) had co-existing dermatological disorders.

## Discussion

It may commonly been remarked that dermatology is usually considered as an outpatient specialty associated with low mortality [15]. This notion could lead to less dermatological attention given to hospitalized patients by some of their attending physicians. Less dermatologic attention paid to admitted patients may allow most of the skin diseases to run a chronic course with significant effects on the general health as well as the quality of life of the affected individual.

Certain systemic disorders can be suspected through cutaneous symptoms and signs. This hospital-based cross sectional study has described the magnitude of co-existing dermatological disorders among patients admitted to medical wards of a national consultant hospital. Almost all forms of skin diseases (infectious, non-infectious, neoplastic, non-specific rashes) were encountered although at different frequencies. When specific types of dermatological disorders were analyzed, the most common were fungal infections (18%), bacterial infections (7.1%), and viral infections (5.3%). Many community based studies conducted in developing tropical countries have described infectious dermatological disorders, especially fungal and bacterial infections as being the most commonly encountered [1-3,11].

The pattern of co-existence between dermatological and other medical conditions in our study demonstrated that, over three-quarters of patients with PPE, seborrheic dermatitis, oral candidiasis, cutaneous warts and psoriasis had HIV/AIDS. On the other hand, all patients with Kaposi's sarcoma (11/11; 100%) were HIV infected. HIV infection has been reported to cause its greatest impact on the skin whereby, today over fifty different types of HIV-related skin diseases have been documented [14-16]. Various studies on HIV related mucocutaneous manifestations conducted worldwide, have documented HIV related skin diseases as being very common [14-17]. In this study it has also been observed that, a wide variety of systemic diseases co-existed with dermatological conditions. Systemic diseases which demonstrated high frequency of dermatological disorders (>50%) included diabetes mellitus, chronic kidney disease, hematological disorders hepatic diseases and neurological diseases.

The finding of cellulitis in only 1.5% of admitted patients in our study, while cellulitis tends to be one of the frequent causes of admission in developed countries may not be surprising since in our set up, mild forms of cellulitis would normally be managed at peripheral hospitals while severe cellulitis which is usually associated with dermal necrosis and fasciitis would be admitted to surgical (and not medical) wards for surgical interventions such as surgical toilet, slouphectomy and even skin grafting.

The prevalence of dermatological disorders at pre-admission (referral) and admission periods, was grossly underestimated for all disorders except for Kaposi's sarcoma which was over-diagnosed. Most skin diseases were overlooked by the referring and admitting doctors. In many areas, health professionals have been reported as being unaware of the burden of dermatological diseases [5]. Health workers tend to overlook or even ignore skin diseases despite the fact that some of them form important signs or symptoms of the underlying internal diseases. This apparent 'unawareness' attitude has been partly attributed to inadequate knowledge of dermatological disorders among clinicians [5]. On the other hand, it could also be speculated that the majority of patients presenting to hospitals for various diseases, may not complain about their accompanying dermatological problems (which would have enabled clinicians to easily detect them), probably due to the assumption that skin diseases are a mere cosmetic nuisance, not associated with any serious suffering. All these factors may lead to delays in diagnosing the underlying serious and even life threatening systemic diseases.

## Conclusion

Dermatological disorders are very common and diverse among patients admitted to medical wards but they are rarely documented as primary or additional diagnoses by their referring or admitting physicians. Dermatological education should be emphasized to the health care workers to improve their knowledge.

## Study limitation

Lack of a specialized dermatology laboratory led to failure to make some confirmatory tests like virological identification of viral infections, immunofluorescent microscopy for blistering diseases and antinuclear antibody tests for connective tissues disorders.

## Competing interests

The authors declare that they have no competing interests.

## Authors' contributions

YMM conceived the study, supervised data collection and prepared the final manuscript as a corresponding author. PNFC participated in study design, conducted data collection and prepared the preliminary manuscript. All authors read and approved the final manuscript.

## Pre-publication history

The pre-publication history for this paper can be accessed here:

http://www.biomedcentral.com/1471-5945/11/8/prepub
